# Facile synthesis of a ZnO nanoplate/Ag nanoparticle hybrid as a highly sensitive SERS substrate for indigo carmine detection

**DOI:** 10.1039/d4ra08973a

**Published:** 2025-04-23

**Authors:** Thi Thu Ha Pham, Ngo Thi Lan, Tran Thu Trang, Nguyen Dac Dien, Truong Xuan Vuong, Thi Thu Thuy Nguyen, Tran Thi Kim Chi, Tran Thi Huong Giang, Nguyen Van Hao, Pham Thi Nga, Dong Thi Linh, Xuan Hoa Vu

**Affiliations:** a Faculty of Chemistry, TNU-University of Sciences Tan Thinh Ward Thai Nguyen City 24000 Vietnam; b Institute of Science and Technology, TNU-University of Sciences Tan Thinh Ward Thai Nguyen City 24000 Vietnam hoavx@tnus.edu.vn; c Faculty of Occupational Safety and Health, Vietnam Trade Union University 169 Tay Son Street, Dong Da District Ha Noi City 10000 Vietnam; d Institute of Materials Science, Vietnam Academy of Science and Technology 18 Hoang Quoc Viet Road, Cau Giay District Ha Noi City 10000 Vietnam; e Faculty of Secondary School, Hoa Lu University 2 Xuan Thanh Street, Ninh Nhat Commune Ninh Binh City Vietnam; f Faculty of Fundamental Sciences, TNU-University of Technology 666 3/2 Road Thai Nguyen City 24000 Vietnam

## Abstract

This work presents the utilization of a hydrothermal treatment and a reduction reaction to synthesize a heterogeneous ZnO nanoplate (NPl)/Ag nanoparticle (NP) nanostructure for application in surface-enhanced Raman scattering (SERS). Under hydrothermal conditions, at 180 °C and 20 h, ZnO NPls with a thickness of 40 nm and edgewise size of 200 nm × 350 nm were prepared from precursors containing zinc acetate (CH_3_COO)_2_Zn and sodium hydroxide (NaOH). Then, Ag NPs with an average diameter of 17 nm were deposited onto the surface of the ZnO NPls by reducing AgNO_3_ using trisodium citrate (TSC). The structural, morphological, and compositional behaviors of the prepared heterostructure were analyzed using X-ray diffraction (XRD), scanning electron microscopy (SEM), transmission electron microscopy (TEM), high-resolution transmission electron microscopy (HRTEM), and energy dispersive X-ray spectroscopy (EDS). The optical properties of the as-prepared products were analyzed using Raman, ultraviolet-visible (UV-Vis) absorption and Fourier transform infrared (FTIR) spectroscopies and photoluminescence (PL) technique. Results confirmed the formation of a ZnO NPl/Ag NP heterostructure, with the Ag NPs adhering to the surface of the 2D semiconducting ZnO NPls. The SERS signal from the chemisorbed indigo carmine (IC) molecules on the ZnO/Ag surface was observed at various concentrations between 5 × 10^−9^ M and 10^−4^ M. The produced SERS substrate demonstrated superior SERS performance in detecting IC, with a low limit of detection (LOD) of 5 × 10^−9^ M, a high enhancement factor (EF) of 1.57 × 10^5^, and good uniformity with a relative standard deviation (RSD) of 3.6%. Raman scattering signals from IC adsorbed on this ZnO/Ag heterostructure showed a significant enhancement compared with those of the same molecules adsorbed on a glass substrate. The surface-enhanced Raman scattering of ZnO/Ag was owing to the hotspots at the Ag NPs and effective charge transport among plasmonic Ag NPs, semiconducting ZnO NPls, and the IC molecules. The most captivating aspect of this study is that the molecular structure of IC was compared using computational and experimental methods; in particular, density functional theory (DFT) calculations using the B97 (d,p) basis set were performed to obtain the optimized geometric structure and frontier molecular orbital of IC molecules. This study provides definitive experimental validation underpinning the phenomenon of SERS on metal oxide semiconductor/noble metal hybrids, which can effectively enhance Raman signals owing to the synergistic action of the electromagnetic (EM) and chemical (CM) mechanisms.

## Abstract 2

Hydrothermal treatment and a reduction reaction to synthesize a heterogeneous ZnO nanoplate (NPl)/Ag nanoparticle (NP) nanostructure for application in surface-enhanced Raman scattering (SERS).

## Graphical abstract

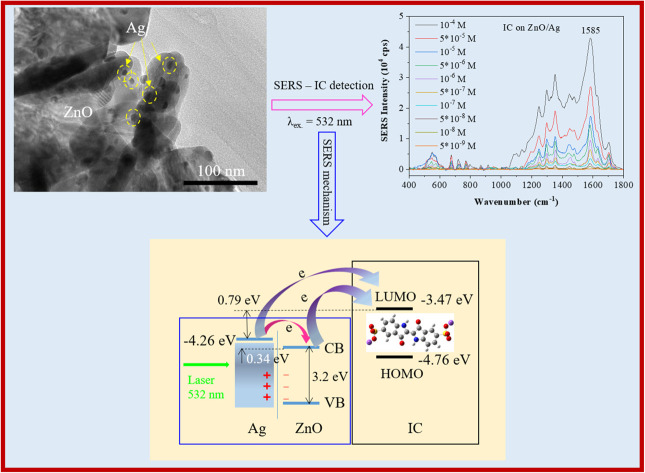

## 1. Introduction

In the last few decades, a sensitive vibrational spectroscopy technique called surface-enhanced Raman spectroscopy (SERS) has been widely studied for detecting organic dye molecules at extremely low concentrations. This phenomenon was first observed by Fleischmann in 1974 when molecules were adsorbed onto metallic nanostructures owing to the plasmonic effect on their surface.^1^ Localized surface plasmon resonance (LSPR) of noble metal nanoparticles, such as gold (Au), silver (Ag), and platinum (Pt), enhances the incident irradiation in the visible to near-infrared (NIR) range.^2^ The strong Raman scattering signals from the plasmonic nanoparticles enable the detection of the adsorbed molecules even at trace levels. SERS has emerged as a powerful tool in semiconductor sensors, providing information on the concentration of target molecules. The scope of SERS-active substrates extends beyond noble metals (Ag and Au)^3,4^ and transition metals (Pt, Pd, Fe, Co, and Ni),^5–8^ as nonmetallic materials, such as In and As,^9^ have also been reported. The mechanism responsible for signal enhancement in semiconductor-based SERS substrates is still not thoroughly investigated. In recent years, semiconductor structures with reduced dimensions have exhibited unique properties associated with quantum confinement, and accordingly, promising potential applications in electrochemical sensing,^10^ gas sensing,^11^ and as SERS substrates^12^ Various metal oxides, such as TiO_2_,^13^ Cu_2_O,^14^ CuO,^13^ and Fe_2_O_3_,^15^ have been investigated in SERS applications. Among them, ZnO serves as a highly adaptable semiconductor material with a wide direct bandgap (*E*_g_ = 3.2 eV), large exciton binding energy (60 meV) and relatively easily modifiable electronic and optical properties.^16^ ZnO also exhibits numerous attractive features, such as facile fabrication, low cost, and non-toxicity. Consequently, it is used as a gas sensor,^17^ SERS substrate,^18^ biosensor,^19^ luminescent material,^20^ and photocatalyst.^21^ ZnO presents certain limitations in real-world SERS applications, particularly due to its inadequate stability and reproducibility. Incorporating plasmonic metals and semiconductor nanostructures to form heterostructures as potential candidates for SERS applications has recently attracted massive attention.^22^ ZnO has been hybridized with noble metals (Ag, Au) to improve SERS performance, including sensitivity and selectivity in detecting probe molecules.^23,24^ The higher Fermi level of Ag compared with ZnO at the ZnO/Ag interface drives electron transfer from Ag to ZnO, leading to electron accumulation in ZnO. The induced electromagnetic field created near the heterojunction and the incident light synergistically form an amplified local field. Therefore, ZnO/Ag is a promising SERS substrate.^25–27^ As detailed in our past work, coating ZnO nanoplates with silver nanoparticles facilitates the identification of MB molecules even at low concentrations by analyzing the Raman spectral bands.^28^ By depositing Ag NPs on other semiconductors, such as Cu_2_O microcubes^29^ and ZnO nanoplates,^24^ the adsorption of species like methyl orange (MO) or methyl red (MR) has been observed on the semiconductor surface even at low concentrations. Smirnov *et al.* decorated Ag NPs on ZnO nanoflowers to detect R6G (rhodamine 6G).^30^ Zeng *et al.* decorated ZnO nanotower arrays with cubic and tetrahedral Ag NPs to fabricate SERS substrates for detecting R6G, demonstrating an EF of 6.9 × 10^13^ and LOD of 10^−18^ M.^25^ Wang *et al.* synthesized a ZnO/Ag heterogeneous nanostructure as an SERS substrate for detecting R6G with a LOD of 10^−8^ M and EF of 7 × 10^8^.^26^ For this reason, in this work, we continued investigating ZnO nanoplate/Ag nanoparticle hybrids in detecting IC molecules. This was inspired by the capability of the SERS technique in detecting surface species, contaminants, and adsorbates, with the Ag-overlayer technique being particularly responsive to minute quantities of organic compounds.^14,31,32^ Semiconductor structures with reduced dimensionality have gained popularity in recent years owing to their exceptional properties, which originate from the quantum confinement effect.^33–35^

Effluents from textile mills comprise toxic chemicals, dyes, pigments, and various organic compounds that are resistant to degradation in aquatic environments. They tend to accumulate in living organisms and harm aquatic life by inhibiting the growth of aquatic phototrophs. They are carcinogenic, mutagenic, and teratogenic agents that cause freshwater contamination.^36,37^ The impact of dyes can manifest as various diseases, including impairment of the kidney, brain, liver, and central nervous system.^38,39^ Azo dyes are currently used on a large scale in the textile industry. Azo dyes are defined by the presence of a nitrogen–nitrogen double bond (N=N) connected to benzene rings, which provide color.^40^ These compounds are known to have various adverse effects on human health, including the development of cancer, chromosomal abnormalities in cells, splenic carcinomas, hepatocarcinomas, renal damage, fever, and cramps.^41^

**Fig. 1:**
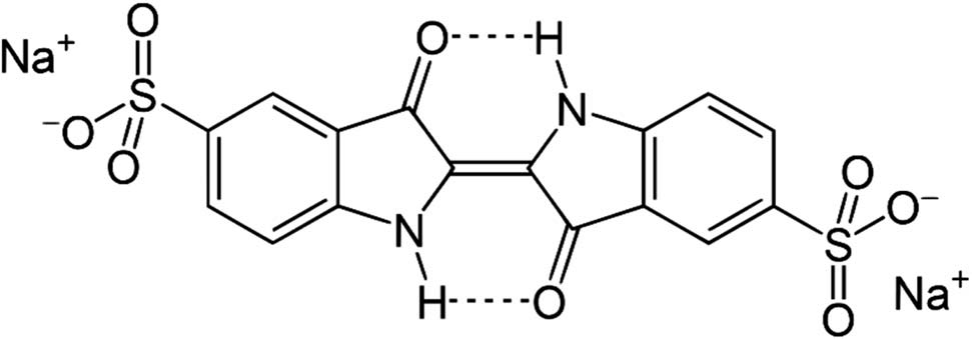
Molecular structure of indigo carmine (IC).

Indigo carmine (IC), also called acid blue 74, indigotine, or E132, belongs to the class of indigoids, with the chemical formula C_16_H_8_N_2_Na_2_O_8_S_2_ and chemical name disodium 5,5′-[(2-(1,3-dihydro-3-oxo-2*H*-indazol-2-ylidene)-1,2-dihydro-3*H*-indol-3-one)] disulfonate. Its molecular structure is presented in Fig. 1. Hydrogen bonding can take place between the hydrogen atom of the N–H bonds and the oxygen atom of the C=O bonds. IC appears as a dark blue powder and is an artificial food colorant and industrial blue pigment. It is also present in processed food items (such as confectionery, patisserie products and biscuits) and pharmaceutical goods.^42^ IC may cause nausea, vomiting, high blood pressure, skin rashes, breathing problems, and allergic reactions. It has been utilized as a chemopreventive agent but can produce mutagenic effects. IC can be used for the detection of adenomas, as an antipsychotic drug, and for the measurement of ozone concentration, but can generate brain tumors.^43^ Owing to its high water solubility, the risk of toxicity and detrimental effects on the ecosystem and biodiversity, it is important to determine even low IC concentrations. This compound functions as a donor–acceptor pigment, with the amine groups serving as electron donors and the carbonyl groups as electron acceptors.^44^

Viviana and colleagues combined density functional theory (DFT) calculations with SERS experiments, relating the Raman frequencies exhibited by an indigo molecule when adsorbed onto the surface of silver.^45^ The findings demonstrated that the indigo-Ag16 structure with 16 silver atoms offers a more comprehensive depiction compared to structures incorporating only one or two silver atoms bound to the indigo molecule. An evaluation of the utilization of SERS for the semi-quantitative assessment of indigo was conducted using citrate-reduced silver colloids, with specific adjustments to the experimental procedures to achieve the optimum SERS signal strength. The SERS spectral profile acquired using silver nanoparticles exhibited distinctions from the Raman profile, suggesting that the interaction between indigo and silver falls within the realm of intermolecular interactions.

Roy *et al.* used the Gaussian 09 software to calculate the density functional theory (DFT) and used the 6-311++G(d, p) basic set to optimize the molecular structure and estimate the vibrational frequencies of methylene blue molecules adsorbed on nanocolloidal gold particles.^46^ N. Peica and W. Kiefer used DFT to understand the optimized geometry and the vibrational wavenumbers of the indigo carmine dye. They used the Gaussian 03 program to optimize the geometry and calculate the normal mode of IC and employed silver colloidal particles to detect indigo carmine (IC) at a low concentration of 0.03 × 10^−9^ M. The 6–311++G** Pople split valence and LANL2DZ basis sets were used to optimize the molecular geometries.^47^ Pagnacco determined micro-scale concentrations of IC through the clock reaction or the Briggs–Rauscher oscillatory kinetic method.^44^

To the best of our knowledge, very few works have been done using ZnO/Ag-based SERS substrates to detect indigo carmine. This is the first study in which SERS is employed to directly investigate the adsorption of IC molecules on ZnO/Ag nanostructures, adding to the novelty of this research. The Raman signal from the ZnO/Ag substrate was improved by several orders of magnitude compared with that from the Ag substrate, and ZnO/Ag presented extremely high sensitivity to IC molecules. In this study, ZnO/Ag nanostructures synthesized using hydrothermal and reduction routes were applied in SERS. The produced nanostructures were analyzed by scanning electron microscopy (SEM), transmission electron microscopy (TEM), high-resolution TEM (HRTEM), photoluminescence (PL), X-ray diffraction (XRD), energy X-ray dispersive spectroscopy (EDS), Fourier Transform Infrared (FTIR), and ultraviolet-visible (UV-Vis) absorption spectroscopy to characterize the crystallographic, morphological, optical properties. Herein, Raman scattering was employed to investigate the adsorption of IC molecules on the ZnO nanoplate/Ag nanoparticle hybrid directly. The performance of the produced SERS substrate was evaluated based on its LOD, EF, and relative standard deviation (RSD). In addition, this report delves into the underlying mechanism responsible for the signal enhancement of the ZnO/Ag-based SERS substrates. The proposed SERS mechanism may be helpful in gaining insights into the charge transfer processes between the adsorbates and semiconductor materials. Density functional theory (DFT) calculations of the Raman spectrum were also used to enhance the geometrical optimization of indigo carmine.

## 2. Experimental section

### 2.1. Materials

Zinc acetate (CH_3_COO)_2_Zn, sodium hydroxide (NaOH), trisodium citrate dihydrate (TSC, Na_3_C_6_H_5_O_7_·2H_2_O), silver nitrate (AgNO_3_), indigo carmine (C_16_H_8_N_2_Na_2_O_8_S_2_), and absolute ethanol (C_2_H_5_OH) of analytical grade were purchased from Merck Chemical Co. and used without further purification. Purified water was utilized in all experiments.

### 2.2. Fabrication of ZnO NPls

ZnO nanoplates were synthesized using a hydrothermal method, as described in the literature.^28^ Briefly, 83 mL of a NaOH solution (1.5 M) was added dropwise into 50 mL of a (CH_3_COO)_2_Zn solution (0.5 M) at room temperature under stirring for 15 minutes to form a transparent mixture. Subsequently, the as-prepared sol was kept at 180 °C for 20 h in a stainless-steel autoclave and then naturally cooled to room temperature. The hydrothermal product was rinsed with deionized water and absolute ethanol three times and dried at 80 °C for 24 h.

The overall reaction of the system can be represented by the following equations:

1 (CH_3_COO)_2_Zn + 2NaOH → Zn(OH)_2_ + 2CH_3_COONa

2
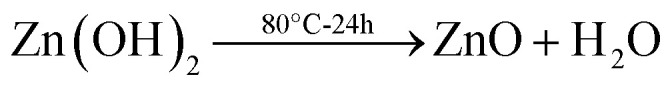


### 2.3. Synthesis of ZnO/Ag heterogeneous nanomaterials

The ZnO nanoplates were surface-modified with Ag nanoparticles as follows: the ZnO NPls powder (60 mg) was suspended in 20 mL of deionized water, and the mixture was stirred continuously for 15 min. Then, 20 μL of a trisodium citrate (TSC) solution (0.6 M) and 100 μL of an AgNO_3_ solution (0.2 M) were added into the ZnO suspension and stirred for 2 h. TSC reduced Ag^+^ ions to form Ag^0^ metal as per the following reaction:^48^

3 4AgNO_3_ + Na_3_C_6_H_5_O_7_ + 2H_2_O → 4Ag^0^ + C_6_H_5_O_7_H_3_ + 3NaNO_3_ + HNO_3_ + O_2_↑

The yellow-brown product was subjected to filtration, followed by multiple rinses with purified water and absolute ethanol, and then dried at 80 °C overnight to obtain ZnO NPls modified with Ag NPs. To obtain pure Ag NPs, the reaction depicted by eqn (3) was carried out in the absence of ZnO NPls.

### 2.4. Sample characterization

The crystal structure of the samples was investigated *via* X-ray powder diffraction (XRD) using a Bruker D8 Advance diffractometer (Germany) with a Cu-K_α_ radiation source (wavelength 0.154056 nm) in the theta–2theta configuration and operated at 40 kV. The surface morphology of the samples was studied using a field emission scanning electron microscope (SEM, Hitachi S4800, Japan) operated at 10 kV. The JEM-2010 (JEOL, Japan) instrument was operated at 80 kV and 200 kV to obtain transmission electron microscopy (TEM) and high-resolution transmission electron microscopy (HRTEM) images, respectively. The chemical composition of the samples was assessed by energy-dispersive X-ray spectroscopy on a Hitachi SU 8020 (EDS, Japan) operated at 200 kV. The ultraviolet-visible-near infrared (UV-Vis-NIR) absorption spectra were recorded on a JASCO V770 UV-vis spectrophotometer (Japan) in the wavelength range of 250–800 nm. A fluorescence spectrometer (FLS1000, Scotland) was employed for photoluminescence (PL) spectral analysis. Fourier-transform infrared spectroscopy (FTIR) was carried out on an ALPHA II spectrometer (Bruker, Germany) to confirm the functional groups in the materials. The Raman spectra were obtained using an EnSpectr R532 (USA). The 532 nm radiation from a Nd:YAG laser was used as the exciting source. Data were acquired after 10 s accumulation time.

### 2.5. SERS spectra measurement

To determine the possibility of Raman signal enhancement of the ZnO/Ag material in the presence of indigo carmine (IC), we investigated the signals obtained from glass, ZnO NPls, Ag NPs, and ZnO/Ag substrates. These SERS substrates were made by fabricating a very thin layer of ZnO, Ag, or ZnO/Ag on a glass plate. Then, 5 μL of IC (concentration of 10^−4^ M) was used to cover these SERS substrates before recording the Raman signals. In order to compare, 5 μL of IC (concentration of 10^−4^ M) was dropped on the pristine glass plate. The target molecules in the samples were detected based on the characteristic peaks (fingerprint bands) of inelastic scattering light. A range of IC solutions with concentrations varying from 10^−4^ M to 5 × 10^−9^ M were meticulously concocted in bi-distilled water flasks. Subsequently, 5 μL of the assorted IC solutions were affixed to the ZnO/Ag substrate using the drop-and-dry technique, followed by the acquisition of Raman spectra under ambient conditions. The Raman spectra were recorded on an Xplora Plus MicroRaman spectrometer (France) after 10 s exposure time using a 532 nm Nd:YAG laser as the excitation source for Raman signals. In order to prevent laser-induced sample annealing and alteration, the laser power applied to the sample was maintained at 3.2 mW. Using a 100× microscope objective, the excitation light was focused on a circular spot of diameter around 3.8 μm on the sample surface. Backward scattered light from the volume close to the focal point was the only light that reached the detector, and it was gathered in the range of 400–1800 cm^−1^. The studied volume allowed a focal depth of approximately 1 μm and a lateral resolution of around 10 μm. The Raman signal was contributed by all IC molecules within the collection volume.

In this experiment, to verify the stability and reproducibility of these SERS-active substrates, after the first SERS measurement, the sample was aged for 3 months before the second SERS test. To recover the SERS substrate materials, the centrifugation method was used to separate ZnO/Ag from IC in water and ethanol. The SERS substrate with adsorbed IC was placed in a centrifuge tube, deionized water was added, and the mixture was vortexed to dissolve IC into the aqueous phase. Then, the mixture was centrifuged at 5000 rpm to collect the solid. This process was repeated 2–4 times in water, depending on the initial IC concentration. Afterward, deionized water was replaced with absolute ethanol for a final wash. The recovered solid, free of IC, was dried and reused as the SERS substrate in another run.

### 2.6. Calculational details

Density functional theory (DFT) calculations were carried out using the Gaussian 09 software suite for the geometry optimization of the molecular structure and the estimation of the vibrational frequencies of IC molecules. The Raman spectrum of indigo carmine was obtained using the B97D/cc-pVZT level of theory with the B97 (d,p) basis set. The choice of function was determined based on a prior examination.^44^ Furthermore, multiple potential spins were taken into account for each geometry to ensure the reliability of the optimization process in identifying the ground state.^49^

## 3. Results and discussion

### 3.1. Morphology, size, and composition

**Fig. 2:**
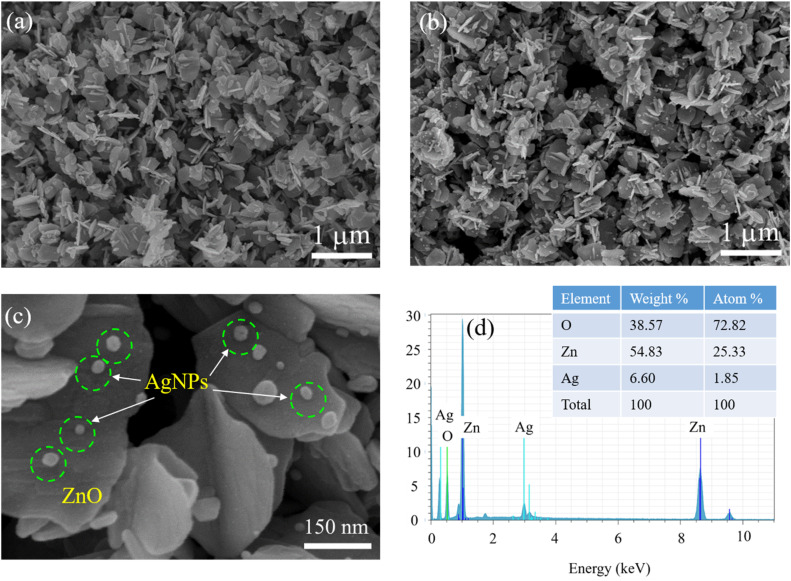
SEM images of (a) ZnO NPls, (b) ZnO/Ag at 20k magnification, and (c) ZnO/Ag at 150k magnification. (d) EDS spectrum of ZnO/Ag.

Fig. 2a and b depict the SEM images of ZnO NPls and the ZnO/Ag hybrid, respectively, at 20k magnification, showing a heightened concentration of consistent ZnO nanoplates with an average thickness of approximately 40 nm, roughly 200 nm in width and 350 nm in length. It is clearly seen that nearly spherical Ag NPs with a narrow size distribution randomly protrude from the surface of the ZnO NPls (the green ring in Fig. 2c). The microscopic images of ZnO NPls and ZnO/Ag hybrid reveal that the Ag NPs had no influence on the morphology of ZnO nanoplates. Fig. 2c displays the SEM image of Ag NPs, providing detailed morphological information at a magnification of 150k. Fig. 2d displays the EDS spectrum, which confirms the existence of Zn, O, and Ag elements in the ZnO/Ag nanostructures, and the composition was 25.33 at% of Zn, 72.82 at% of O, and 1.85 at% of Ag.

**Fig. 3:**
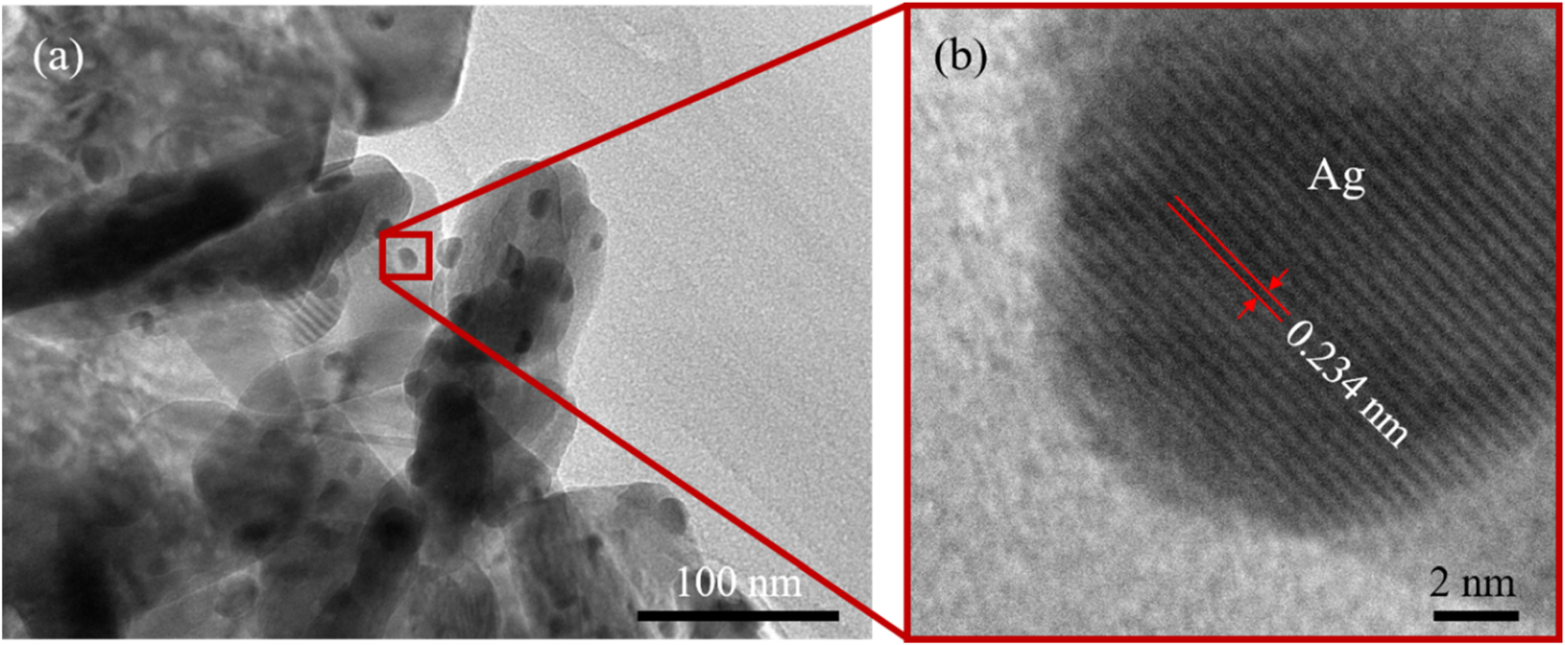
(a) Typical TEM and (b) HRTEM images of ZnO/Ag.

The TEM image of the ZnO/Ag hybrid structure (Fig. 3a) revealed numerous silver nanoparticles (Ag NPs) uniformly dispersed over the surface of the ZnO NPls, facilitating enhanced conductivity for electron transport between ZnO and Ag. The size of the Ag NPs was estimated to be about 17 nm. The TEM image illustrates the overlapping of ZnO NPls, which forms a three-dimensional network structure.

The high-resolution TEM (HRTEM) image shown in Fig. 3b reveals that the distance between the adjacent lattice fringes corresponding to the [111] plane of Ag was nearly 0.234 nm. This lattice constant was determined based on the *d*-spacing of Ag using the below equation:4
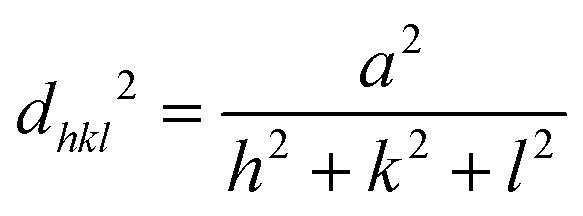


The lattice parameter of silver (Ag) was determined to be *a* = 0.405 nm.

### 3.2. Crystalline structure

**Fig. 4:**
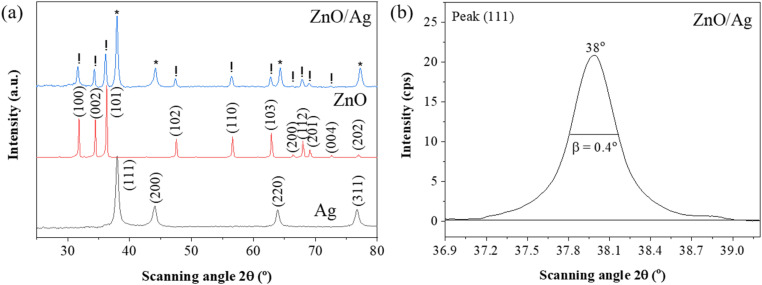
(a) XRD patterns of ZnO NPls, Ag NPs, and ZnO/Ag hybrid. (b) Full-width at half-maximum (FWHM) of the most intense diffraction peak.

Fig. 4a shows the XRD patterns of the ZnO nanoplates, Ag nanoparticles, and ZnO/Ag hybrid. The diffraction peaks of ZnO marked by the ! symbol, including the dominant XRD peaks at 2*θ* = 32°, 34°, 36° and the smaller peaks at higher 2*θ* values, confirm the hexagonal wurtzite phase of ZnO (JCPDS card no. 36-1451).^50^ The diffraction peaks of Ag marked by * at 2*θ* = 37.9°, 44°, 63.3° and 76.7° indicate the face-centered cubic (FCC) crystal structure of Ag (ICDD card no. 01-071-3752).^50^ It can be observed that the diffraction peaks are narrow, indicating a good degree of crystallinity in the ZnO sample. The Scherrer formula was utilized to estimate the size of the crystalline domains (*D*) from the half-bandwidth of the X-ray spectral peak:^51^5
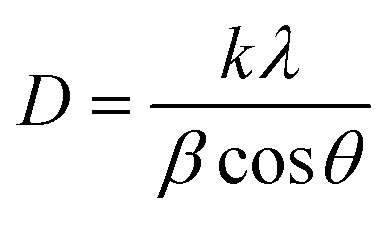


The formula incorporates parameters, such as the X-ray wavelength (*λ* = 0.154056 nm), the Scherrer constant (*k* = 0893), the full-width at half-maximum (FWHM) denoted by *β* in radians, and the Bragg diffraction angle (*θ*). The achieved *D* values of ZnO NPls, Ag NPs and ZnO/Ag at 36.3°, 38°, and 38° corresponded to crystallite sizes of 41.5 nm, 16.7 nm, and 20.8 nm, respectively (Fig. 4b).

### 3.3. UV-vis spectra

**Fig. 5:**
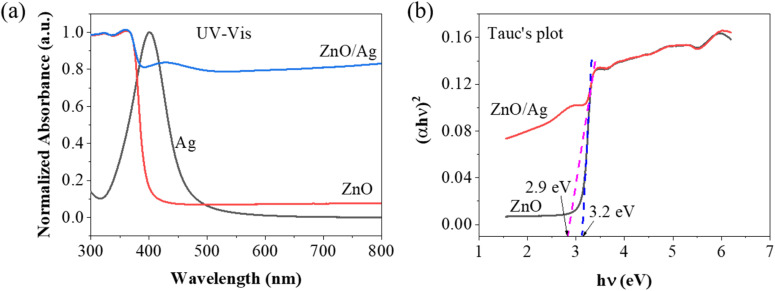
(a) Normalized UV-Vis absorption spectra of Ag, ZnO/Ag, and ZnO nanoplates; (b) Tauc plots of ZnO and ZnO/Ag samples, illustrating the bandgap energy.

The UV-vis spectra were recorded to investigate the optical properties of the ZnO nanoplates and ZnO/Ag hybrid. Fig. 5a clearly illustrates the UV-vis spectra of pure Ag NPs, ZnO NPls and the ZnO/Ag hybrid. A wide optical absorption band below 400 nm originating from the band–band transition of the ZnO nanocrystals was observed in both ZnO and ZnO/Ag samples. Nevertheless, the absorption spectrum of the ZnO/Ag hybrid displayed a broad band with a central wavelength of 428 nm, which can be attributed to the surface plasmon resonance (SPR) of Ag metallic nanoparticles. The UV-vis spectrum of pristine Ag NPs showed an absorbance band at 400 nm, indicating the elemental silver form.

The optical bandgaps of ZnO and ZnO/Ag samples were calculated using the Tauc equation:

6 (*αhν*)^2^ = *A*(*hν* − *E*_g_)

where *α* is the absorption coefficient, *h* = 6.625 × 10^−34^ Js is Planck's constant, *ν* is the incident photon frequency (Hz), *hν* is the photon energy, *A* is a constant, and *E*_g_ is the bandgap energy. This equation is applicable to semiconductors with a direct allowed transition. The optical bandgap of the samples was calculated by extrapolating the linear portion in the Tauc plots of (*αhν*)^2^ against *hν* presented in Fig. 5b. The calculated bandgaps of ZnO and ZnO/Ag were 3.2 eV and 2.9 eV, respectively, indicating that the bandgap of ZnO decreased after the addition of Ag. This value aligns closely with the findings reported previously.^45^

**Fig. 6:**
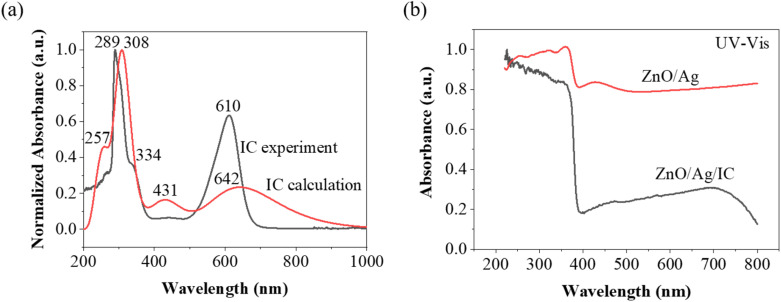
(a) Absorption spectra of IC obtained *via* experiment and calculation and (b) comparison of the absorption spectra of ZnO/Ag and ZnO/Ag/IC.

Fig. 6a shows the absorption spectrum of IC consisting of 4 characteristic peaks in the range of 200–1000 nm. The original blue color of IC corresponds with the dominant signal at 610 nm, which falls in the visible spectrum. The peak at 289 nm represents an amino group. The carbonyl group is responsible for the signal at 257 nm, while the aromatic ring resonance results in a peak at 334 nm. Once again, this outcome emphasizes the enhancement of the Raman scattering of ZnO in ZnO/Ag. Fig. 6b presents the absorption spectrum of IC adsorbed on the ZnO/Ag surface. These spectra show the effect of the adsorbed IC molecules on the optical properties of the ZnO/Ag nanostructure. The occurrence of an absorption edge at 700 nm can be attributed to a notable interaction between the adsorbed molecules and the ZnO/Ag substrate.

### 3.4. Optical properties

**Fig. 7:**
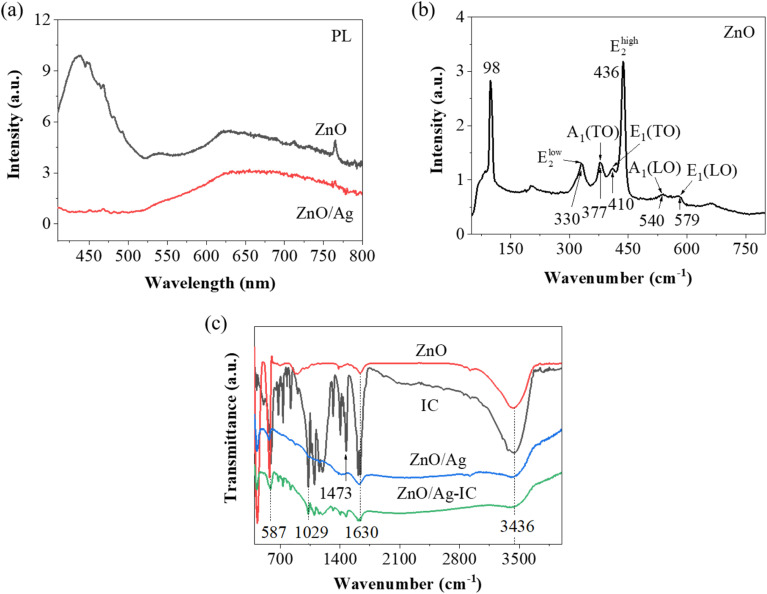
(a) Photoluminescence spectra of ZnO nanoplates and ZnO/Ag hybrid excited at 305 nm. (b) Raman spectrum of ZnO in the range of 50–800 cm^−1^. (c) FTIR spectra of ZnO, ZnO/Ag, IC, and ZnO/Ag-IC.

The optical characteristics of ZnO/Ag were examined through photoluminescence analysis in comparison with pure ZnO, and an evident difference in fluorescence intensity was detected between the two spectra, as illustrated in Fig. 7a. The fluorescence emission of ZnO in the ZnO/Ag composite sample was significantly suppressed in contrast to pure ZnO under excitation using a 305 nm laser. It is widely acknowledged that fluorescence and Raman spectroscopy represent contrasting phenomena. The fluorescence emission is associated with a radiative process, while Raman scattering corresponds to a non-radiative process. Consequently, the fluorescence quenching of ZnO in the ZnO/Ag composite indicates an increase in non-radiative processes.^26^

For a more in-depth analysis of the structures of the synthesized samples, Raman spectroscopy serves as a potent non-destructive characterization technique to explore the vibrational structure of oxides or hybrid materials. It is based on the inelastic scattering of photons by phonons in the system. Raman scattering, mostly in the visible range, is primarily governed by the electrons involved in the scattering process.^52^ The photon–phonon interaction stimulates the material to reach an intermediate state by generating electron–hole pairs. Subsequently, the e–h pairs are redirected to another state by either emitting or absorbing a phonon through the electron–phonon interaction. When an electron and a hole recombine, a scattered photon with a lower or higher energy than that of the incident photon is emitted, while the electronic state of the material remains the same.^53^ The hexagonal lattice of the ZnO wurtzite structure consists of two interpenetrating sub-lattices of the Zn^2+^ and O^2−^ ions, where each O^2−^ ion is surrounded by a tetrahedron of Zn^2+^ ions and *vice versa*. The *c*-axis is the hexagonal vertical axis of the ZnO polar symmetry.^54^

Fig. 7b shows the vibrational modes of the pristine ZnO nanoplates, showing many peaks in the range of 200 to 800 cm^−1^. The most intense line is located at 436 cm^−1^, along with a band at 330 cm^−1^; weaker ones are at 377, 410, 540, and 579 cm^−1^. A prominent background supports the peaks and can be attributed to the semiconductor overtone and combination bands.^55^*A*_1_ represents the lattice vibration of the atoms parallel to the *c*-axis. *E*_1_ corresponds to the lattice vibrations of the atoms oriented perpendicular to the *c*-axis. The displacement of the Zn sub-lattice in relation to the O sub-lattice results in a coherent oscillating polarization; thus, the *A*_1_ and *E*_1_ phonons are classified as polar modes. The *A*_1_ mode is divided into transverse optical (TO, at 377 cm^−1^) and longitudinal optical (LO, at 540 cm^−1^) phonons. Similarly, the *E*_1_ mode encompasses transverse optical (TO, at 410 cm^−1^) and longitudinal optical (LO, at 579 cm^−1^) phonons. The most intense peaks are attributed to the *E*_2_ modes. Vibrations at high frequencies (such as *E*_2_^high^, at 436 cm^−1^) and low frequencies (such as 330 cm^−1^) correspond to movements within the O and Zn sub-lattices, respectively. Within each sub-lattice, adjacent ions move in opposite directions, resulting in the cancellation of induced polarization, and hence, these modes are termed non-polar modes.

FTIR vibrational spectra are valuable for elaborating the structure and compositional characteristics of materials. The infrared spectra of ZnO, ZnO/Ag, IC, and ZnO/Ag-IC are shown in Fig. 7c. Four strong peaks can be observed in the FTIR spectrum of IC. The intense peak at 1630 cm^−1^ is due to the stretching vibration of the conjugated system of C=C and C=O groups. The shorter wavelength peak at 1029 cm^−1^ is specific to the stretching vibration of C–H in the aromatic ring. The 587 cm^−1^ peak is assigned to C–C stretching, and the 3436 cm^−1^ peak is attributed to the stretching vibration of the N–H bond.^49^ The band at 1473 cm^−1^ is ascribed to the C–H bending vibration and S=O stretching vibration of the sulfonate group. The results of indigo carmine are consistent with a previous study.^56^ The broad absorption bands at 3436 cm^−1^ in ZnO/Ag denote the stretching vibrations of –OH. The successful adsorption of IC dye is confirmed by the decreasing intensity of the bands at 1473 and 1029 cm^−1^, which demonstrates the existence of S=O and S–O stretching in the SO_3_ groups of the IC molecules.^57^

### 3.5. Geometry optimization of the indigo carmine molecule

DFT calculations based on the Raman spectrum of Indigo carmine were carried out to achieve the geometrical optimization of IC molecules with the B97 (d,p) basis set. The bond lengths and angles of the molecule were optimized to characterize the compound. An IC molecule comprises 38 atoms with 30 normal vibration modes that are categorized under *C*_s_ symmetry. The assignment of the Raman peaks of the IC adsorbed on glass, and the SERS spectra of IC adsorbed on ZnO/Ag and Ag from the experimental results and the computational regimes are presented in Table 1. The vibrational peak at 1577 cm^−1^ slightly shifts to lower energy by 8 cm^−1^ and 3 cm^−1^ for IC adsorbed on ZnO/Ag and Ag surfaces, respectively. These frequency shifts can be attributed to the notably different interactions of adsorbed IC on the different solid surfaces. Due to the lone pair of electrons on the nitrogen atoms, IC is a strong electron donor. Therefore, it is hypothesized that when IC molecules adsorb onto the ZnO/Ag nanostructure, their electrons are likely trapped by the electron-deficient silver atoms. The pronounced band at 1585 cm^−1^ is linked to the chemisorption of IC on the ZnO surface. The strong band at 1698 cm^−1^ in the Raman spectrum is ascribed to the stretching vibration of the 
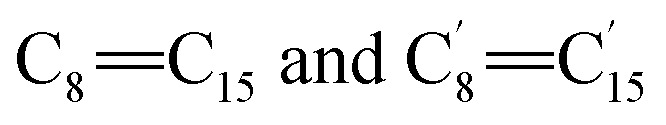
 bonds of both pyrrolidone rings and the symmetrical 
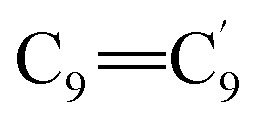
 bridge bond stretching modes between the two phenyl rings.^58^ The medium band at 1621 cm^−1^ is assigned to the in-plane C–H bending mode of the phenyl rings.^59^ The very strong band at 1585 cm^−1^ is assigned to the C_2_=C_3_ and C=O stretching modes of both pyrrolidone rings. The strong band at 1347 cm^−1^ is due to the in-plane N–H and C–H bending modes and the 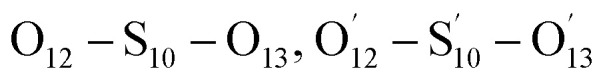 stretching contributions.^60,61^ This band was observed at 1352, 1352, and 1349 cm^−1^ when IC was adsorbed on glass, ZnO/Ag, and Ag, respectively (Fig. 9a–c). The strong peak at 1291 cm^−1^ is assigned to the stretching modes of the 
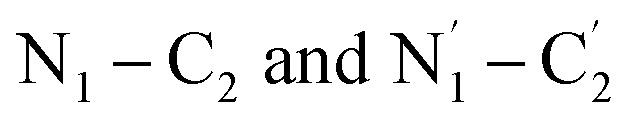
 bonds and the C–C bending mode.^60,61^ The medium band at 1231 cm^−1^ corresponds to the in-plane C–H and C=O bending contributions. The very weak band at 759 cm^−1^ corresponds to the out-of-plane C–H and C–C bending modes. The very weak band at 695 cm^−1^ results from the 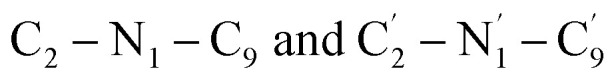 bending modes. The weak peak at 665 cm^−1^ is assigned to the out-of-plane N–H and C–C bending modes of both pyrrolidone rings. The weak signal at 560 cm^−1^ is assigned to the 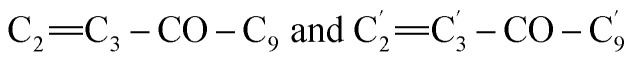 bending modes.^62,63^

**Table 1:** Band assignment for the IC molecule and comparing theoretical DFT calculations with Raman spectra of IC on glass, ZnO/Ag and Ag NPs

Raman calculation (cm^−1^)	IC on glass (cm^−1^)	IC on SERS surfaces (cm^−1^)		Band assignments
		ZnO/Ag	Ag NPs	
560.24	546	550	549	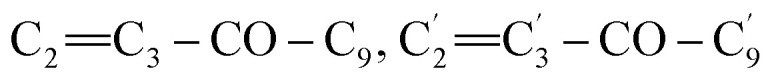 , bending, weak
664.67	669	675	673	C–C bending, weak
				N–H bending, weak
695.15	719	720	722	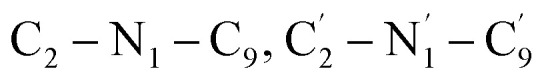 Bending, very weak
759.03	771	772	768	C–H out-of-plane bending, very weak
				C–C out-of-plane bending, very weak
1230.87	1247	1254	1249	C–H in-plane bending, medium
				C=O in-plane bending, medium
1291.06	1296	1297	1296	C–C bending, strong
				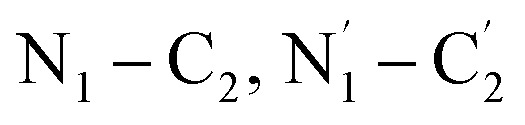 stretching, strong
1346.69	1352	1352	1349	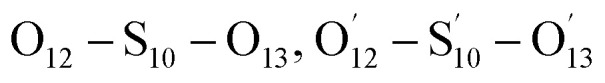 stretching, strong
				N–H in-plane bending, strong
				C–H in-plane bending, strong
1584.96	1577	1585	1580	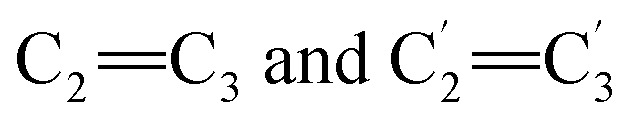 ring Asymmetrical stretching, very strong
				C=O stretching, very strong
1620.87	1629	1627	1630	C–H in-plane bending, medium
1698.25	1701	1703	1699	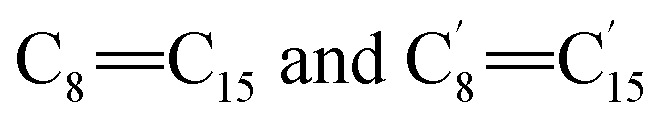 bonds Stretching, strong
				*C* = *C* double bond stretching, strong

**Fig. 8:**
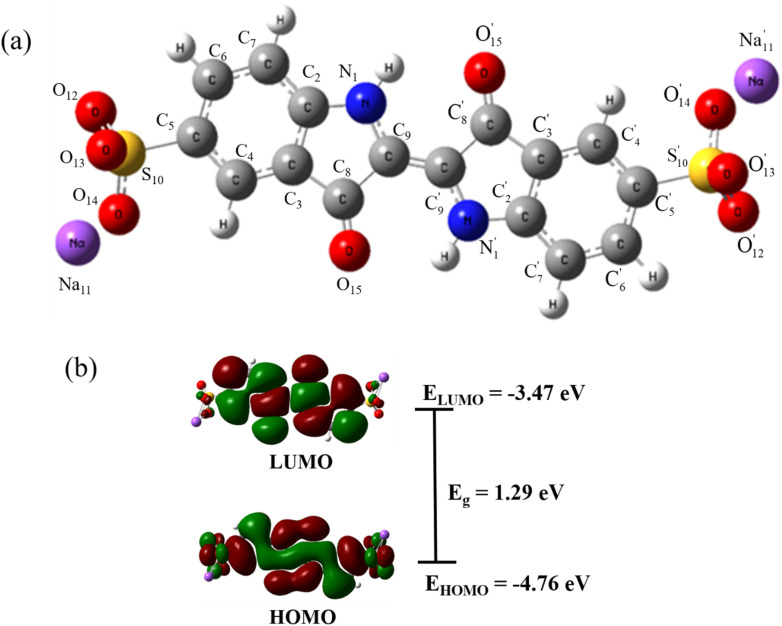
Optimized molecular structure (a) and Frontier molecular orbitals (b) of IC calculated using the B97D/cc-pVTZ level of theory.

Fig. 8a presents an optimized molecular structure that shows the location of the atoms in three-dimensional space. Fig. 8b depicts the frontier molecular orbitals drawn using the visualization software VMD 1.9.3, encompassing the HOMO and LUMO of IC. These orbitals determine the optical and electrical properties, chemical stability, and chemical activity of molecules. According to the calculation, the energies of the HOMO and LUMO of IC were −4.76 eV and −3.47 eV, respectively. Therefore, the difference between HOMO and LUMO was 1.29 eV. This energy gap reflects the ability of the electrons to transit from HOMO to LUMO if they absorb the incident photons with energies higher than this gap. The small energy gap of the IC molecules indicates chemical instability and easy electron transition.

### 3.6. SERS analysis of the ZnO/Ag nanomaterial

**Fig. 9:**
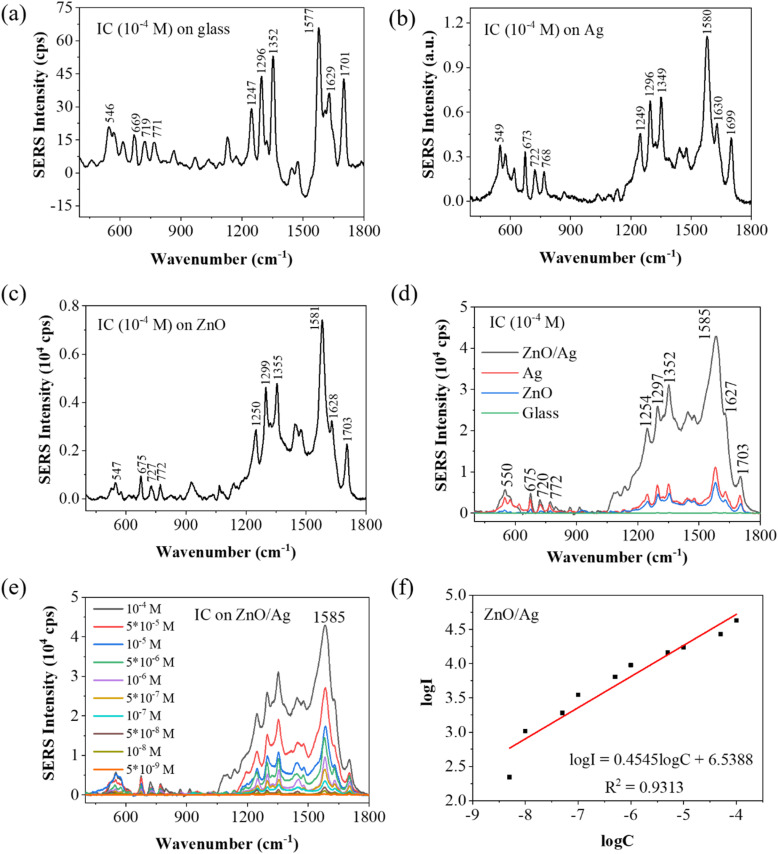
Raman spectra of IC (10^−4^ M) on (a) glass, (b) Ag NPs, and (c) ZnO NPls substrate; (d) comparison of the Raman spectra of IC (10^−4^ M) adsorbed on ZnO/Ag, Ag NPs, ZnO NPls, and a glass substrate; (e) Raman spectra of IC at different concentrations from 5 × 10^−9^ M to 10^−4^ M adsorbed on the ZnO/Ag substrate; and (f) linear relationship between log *I* of the 1585 cm^−1^ peak and log *C* of IC concentration.

To evaluate the SERS activity of the ZnO/Ag substrate, we measured the Raman spectra of IC molecules adsorbed on three types of samples, including bare ZnO nanoplates, pristine Ag nanoparticles, and the ZnO/Ag hybrid coated on a flat glass substrate. The results are depicted in Fig. 9. The SERS spectra of indigo showed the strongest bands at 1573 and 557 cm^−1^, followed by medium bands at 1018 and 401 cm^−1^, a broad medium band at 651 cm^−1^, weak medium bands at 1309 and 460 cm^−1^, broad weak bands at 1487 and 1057 cm^−1^, and weak bands at 1433 and 1194 cm^−1^.^64,65^Fig. 9a shows the normal Raman spectrum of IC adsorbed on a glass plate at a concentration of 10^−4^ M in water (*i.e.*, without Ag, ZnO, or ZnO/Ag). 10 characteristic Raman peaks of the IC dye were identified at 546, 669, 719, 771, 1247, 1296, 1352, 1577, 1629, and 1701 cm^−1^. Fig. 9b shows the SERS spectrum of 10^−4^ M IC adsorbed on the Ag NPs substrate, with dominant peaks at 549, 673, 722, 768, 1249, 1296, 1349, 1580, 1630, and 1699 cm^−1^. Notably, the SERS spectrum of IC adsorbed on Ag differs significantly from the standard Raman spectrum of IC on a glass plate in terms of both the intensity and position of the Raman peaks. Fig. 9c shows the SERS spectrum of 10^−4^ M IC adsorbed on the ZnO NPls substrate, with dominant peaks at 547, 675, 727, 772, 1250, 1299, 1355, 1581, 1628, and 1703 cm^−1^. Fig. 9d shows the SERS spectrum of 10^−4^*M* IC adsorbed on the ZnO/Ag substrate, with dominant peaks at 550, 675, 720, 772, 1254, 1297, 1352, 1585, 1627, and 1703 cm^−1^. The shift of these Raman peaks suggests a significant interaction between the adsorbate and the semiconductor atoms. The SERS spectrum of IC adsorbed on ZnO/Ag was dominated by a strong band at 1585 cm^−1^, which was assigned to the characteristic aromatic ring vibration. The Raman signals of IC adsorbed on the ZnO/Ag substrate were clearly enhanced in comparison with IC on a glass plate. Fig. 9e presents the Raman spectra of IC adsorbed on the ZnO/Ag substrate at different concentrations of IC from 5 × 10^−9^ M to 10^−4^ M. When the IC concentration increased gradually, all ten Raman peaks exhibited a uniform increase in intensity. The ZnO/Ag heterostructure-based SERS substrate demonstrated the greatest Raman signal enhancement.

Most bands of IC adsorbed on the glass plate were also observed for IC absorbed on ZnO NPls, Ag NPs, and ZnO/Ag. Evidently, the SERS intensities of IC from the Ag, ZnO, and ZnO/Ag substrates (Fig. 9b–d) were much higher than those adsorbed on the glass plate (Fig. 9a). The interaction between the IC molecules and surface of the substrates leads to an increase in SERS intensity of all the characteristic peaks in the following order: ZnO < Ag < ZnO/Ag. To characterize the performance of the SERS substrate based on ZnO/Ag, the enhancement factor (EF), limit of detection (LOD), and uniformity were evaluated. The SERS spectra of IC in Fig. 9d allow the evaluation of the SERS strength by comparing the Raman scattering intensity of the IC molecules on the nanostructure surfaces (ZnO, Ag, ZnO/Ag) with those on the glass plate. The enhancement factor (EF) for the 10 peaks at 550, 675, 720, 772, 1254, 1297, 1352, 1585, 1627, and 1703 cm^−1^ were calculated for IC on the ZnO, Ag, and ZnO/Ag substrates using the below formula:^26^7
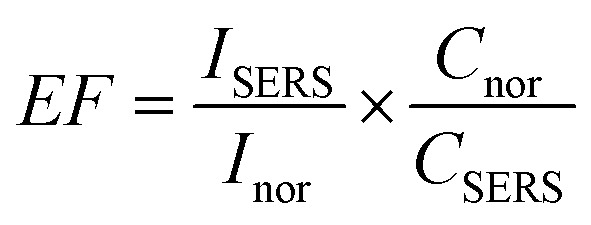
where *I*_SERS_ is the SERS intensity of IC adsorbed on the ZnO, Ag or ZnO/Ag substrate, and *I*_nor_ is the Raman intensity of IC adsorbed on a glass plate under the same experimental condition. *C*_SERS_ = 5 × 10^−9^ M/10^−4^ M is the IC concentration on the SERS substrates, and *C*_nor_ = 10^−4^ M is the IC concentration on the glass plate. The normal Raman measurement was carried out on a plain glass plate with adsorbed IC molecules under the same conditions used for the SERS substrates. The highest EF at the Raman peak 1585 cm^−1^ was calculated to be 1.57 × 10^5^ at an IC concentration of 10^−8^ M. Other EF values of IC adsorbed on the ZnO NPls, Ag NPs, and ZnO/Ag hybrid substrates calculated from different Raman peaks are presented in Table 2. Compared with Ag NPs and ZnO NPls, the ZnO/Ag heterostructure exhibited Raman intensity enhancement of the peak at 1585 cm^−1^ by 3.86 and 5.78 times, respectively. Consequently, IC molecules on the ZnO nanoplate/Ag nanoparticle nanohybrid showed the highest Raman signal compared with those from pristine Ag NPs or ZnO NPls.

**Table 2:** Enhancement factor (EF) of IC molecules (at a concentration of 10^−4^ M) adsorbed on ZnO NPls, Ag NPs, and the ZnO/Ag hybrid substrate

Band (cm^−1^)	Enhancement factor (EF) of SERS substrate		
	ZnO NPls	Ag NPs	ZnO/Ag hybrid
550	102 ± 5.2	168.5 ± 27.3	282.6 ± 41.3
675	55.2 ± 15.7	170.7 ± 28.9	369 ± 80.1
720	85.2 ± 4.3	139.4 ± 26.6	228.8 ± 50.1
772	69.1 ± 3.5	136.8 ± 25.7	202.1 ± 43.2
1254	115.6 ± 18.1	183.9 ± 47	696.5 ± 51.6
1297	102.1 ± 3.5	174 ± 21.7	608.3 ± 28.4
1352	103.6 ± 22.5	153.3 ± 22.8	604.8 ± 15.6
1585	113.4 ± 1.5	176.5 ± 7.3	660.1 ± 34.3
1627	89.5 ± 5.6	97.2 ± 13.7	24.3 ± 2.3
1703	48.3 ± 5.4	144.5 ± 6.5	228.1 ± 23.4

The logarithm of SERS intensity of the 1585 cm^−1^ peak was linearly related to the logarithm of IC concentration in the range of 5 × 10^−9^ M to 10^−4^ M, as shown by the following equation:

8 log *I* = 0.4545 log *C* + 6.5388

and the standard correlation coefficient *R*^2^ = 0.9313 (Fig. 9f). Table 3 summarizes the EF values obtained for different concentrations of IC absorbed on the ZnO/Ag substrate. The EF value decreased as the IC concentration increased from 10^−8^ M to 10^−4^ M. Table 4 shows the comparison of the LOD and EF of ZnO/Ag in detecting various target molecules.

**Table 3:** EF values of the 1585 cm^−1^ peak for different concentrations of IC adsorbed on the ZnO/Ag substrate

IC concentration (M)	EF value	IC concentration (M)	EF value
5 × 10^−9^	62 215.5	10^−6^	14 485.6
10^−8^	156 601	5 × 10^−6^	4394.5
5 × 10^−8^	5936.3	10^−5^	2631.3
10^−7^	53 399.1	5 × 10^−5^	263.1
5 × 10^−7^	19 493.2	10^−4^	660.1

**Table 4:** SERS performance of ZnO/Ag substrates towards various target adsorbate dyes

Morphology	Target dye	EF	LOD	References
Sea-urchin-like	Rhodamine 6 G (R6G)	3 × 10^6^	Not reported	66
Nanodome	Malachite green	10^6^	10^−17^ M	18
Hollow sphere	R6G	3.2 × 10^8^	Not reported	67
Cone-shaped	Polychlorinated biphenyl	3.58 × 10^7^	10^−11^ M	68
Nanoplates	Methylene blue (MB)	6.2 × 10^6^	10^−9^ M	28
Nanoplates	Methyl red (MR)	1.3 × 10^6^	5 × 10^−8^ M	24
Nanotower	R6G	6.9 × 10^13^	10^−18^ M	25
Nanoplates	Indigo carmine (IC)	1.566 × 10^5^	5 × 10^−9^ M	This work

**Fig. 10:**
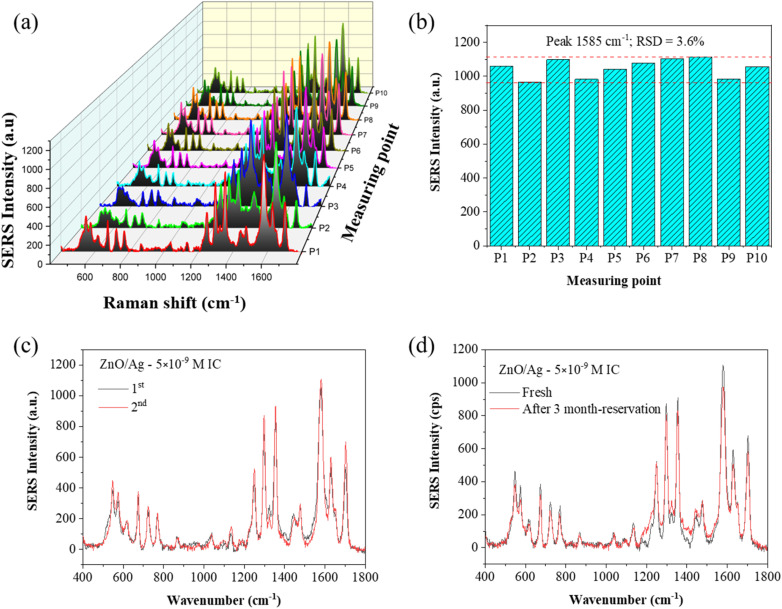
(a) SERS spectra of IC molecules at a concentration of 5 × 10^−9^ M from different spots on the ZnO/Ag substrate; (b) histogram of the peak intensity at 1585 cm^−1^, with a relative standard deviation (RSD) of 3.6%; (c) SERS spectra of 5 × 10^−9^ M IC adsorbed on the ZnO/Ag substrate during two cycles, and (d) stability analysis of ZnO/Ag towards detection of 5 × 10^−9^ M of IC after a 3 months storage time.

The limit of detection (LOD) of the SERS substrate based on ZnO/Ag was analyzed by measuring the Raman spectrum of the IC molecules at a concentration of 5 × 10^−9^ M, as shown in Fig. 10a. Even at the concentration of 5 × 10^−9^ M, all ten Raman peaks of IC could be clearly identified, so this concentration was chosen as the limit of detection of IC using the ZnO/Ag substrate.

Another important feature to be considered is the uniformity of the SERS substrate. Fig. 10a depicts the 10 Raman spectra of IC (5 × 10^−9^ M) absorbed on the ZnO/Ag surface obtained from 10 randomly selected points. The slight fluctuations observed in the peak intensities may be due to the nonuniform adsorption of Ag NPs and IC molecules. The relative standard deviation (RSD) of the peak intensity at 1585 cm^−1^ was estimated to be 3.6% (Fig. 10b). The low RSD value indicates good SERS signal uniformity in terms of both intensity and characteristic bands. With such high homogeneity in SERS characteristics, the SERS substrate based on ZnO/Ag can be considered an excellent candidate for detecting IC molecules. The reproducibility of ZnO/Ag as an SERS substrate was quantified after removing IC from ZnO/Ag and allowing IC re-adsorption at the same concentration (5 × 10^−9^ M). The result seen in Fig. 10c shows the good reproducibility of the proposed SERS substrate. In addition, its stability was evaluated using the best substrate (ZnO/Ag) after a storage period of 3 months, and the Raman enhancement effect remained pronounced. The slight lowering of SERS intensity at the same IC concentration denoted the exceptional stability of ZnO/Ag (Fig. 10d).

### 3.7. SERS mechanism of IC

Although several potential enhancement mechanisms have been suggested, a complete understanding of SERS remains elusive. The current consensus supports the synergistic action of the electromagnetic mechanism (EM) and the chemical mechanism (CM). The electromagnetic mechanism (EM) involves the enhancement of surface plasmon resonance of the metal, so the local electric field at the surface is magnified.^26^ The surface plasmons of Ag NPs and the gap plasmons between neighboring Ag NPs enhance the electric field. Under the action of an electric field, the molecular polarization changes, and the intensity of the Raman signal is intensified. The EM mechanism necessitates the incident radiation to stimulate surface plasmon resonance (SPR), leading to a localized robust enhancement of the electric field at a specific distance from the metal surface. This EM model does not mandate particular bond formation between adsorbates and substrates. EM is denoted as a physical effect due to the physical adsorption of molecules on or near the surface. The level of Raman signal intensity enhancement is determined by the material, shape, size, and density of the metal nanoparticles. The EM mechanism is the most effective when the metal size is in the range of 10–100 nm.^69^ It is well-known that the following equation can assess the plasmon absorption of silver:^70^9
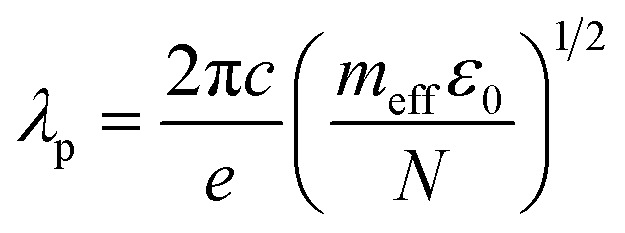
where *m*_eff_ is the effective mass of the free electron in silver, *N* is the electron density of silver, *e* = 1.6 × 10^−19^ C is the electron charge, *c* = 3 × 10^8^ m s^−1^ is the speed of light in the vacuum, and *ε*_0_ = 8.85 × 10^−12^ F m^−1^ is the electrical constant. According to this equation, the plasmon absorption band of silver depends on the electron density *N*; when the electron density of silver decreases, the plasmon adsorption *λ*_p_ increases. The surface plasmon resonance frequency for most semiconductor materials can be calculated using the formula:^71^10
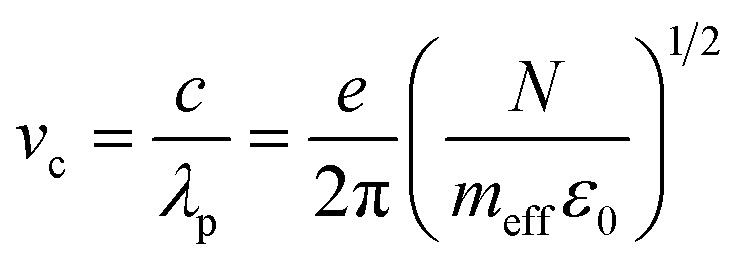


Due to the transfer of electrons from Ag to ZnO, the electron density (*N*) in ZnO increases, and the surface plasmon resonance wavelength (*λ*_p_) is located in the infrared region, which is significantly different from the 532 nm excitation wavelength employed in our experiment. Hence, the electromagnetic mechanism cannot be the main factor responsible for the surface-enhanced Raman signal of IC adsorbed on the ZnO surface.

**Fig. 11:**
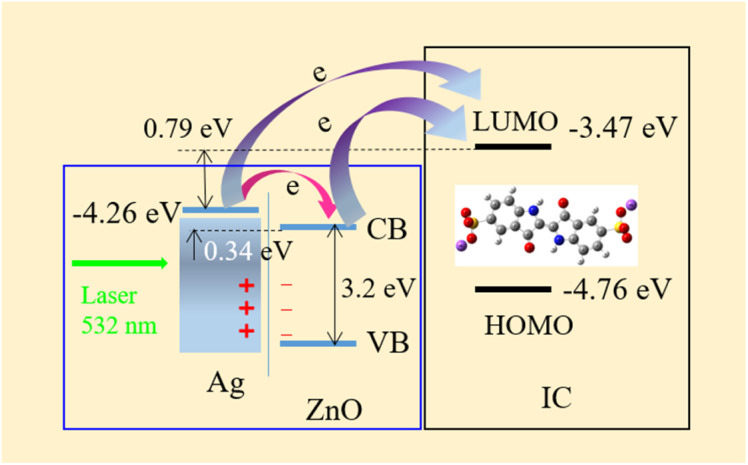
Charge transfer between ZnO/Ag and IC molecules under a 532 nm laser excitation.

The dominant factor driving the SERS signal in this instance is most likely the chemical mechanism (CM), which is associated with the formation of specific adsorbate-metal complexes. The incident photons of the exciting laser light cause a resonant charge transfer (CT) between ZnO, Ag and IC molecules. Both the surface properties of ZnO/Ag and the inherent nature of the IC molecules play significant roles in the charge transfer process. Direct bond formation between the target molecules and the substrate surface is required for the CT process. The robust interaction between the IC molecules and the ZnO/Ag surface, combined with the numerous surface states, promotes charge transfer from ZnO/Ag to the IC molecules. The polarization and Raman scattering cross-section of the adsorbed species are increased. Plasmon-induced hot electrons from the Ag metal are injected into the conduction band (CB) of ZnO under excitation at the wavelength of 532 nm. The Fermi level of Ag is higher than that of ZnO, and the photon energy (2.33 eV) is larger than the energy difference (0.34 eV) between the Fermi level of Ag and the conduction band of ZnO, so the incident photons excite the electrons in Ag to ZnO. At the ZnO/Ag interface, the potential barrier provides the necessary energetic driving force for exciton dissociation. Nevertheless, the 532 nm (about 2.33 eV) laser used as the excitation source in our experiment was not strong enough to facilitate electron transitions from the ZnO valence band (VB) to its conduction band (CB) (*E*_g_ = 3.2 eV). The Raman signal emitted from the IC molecules requires electron excitation at the LUMO level of IC molecules. Taking into account the energy band structure of ZnO/Ag, charge transfer between ZnO/Ag and the IC molecules can occur *via* three distinct modes, as outlined below. The first path involves electron transfer from the HOMO level of the adsorbed IC molecules excited by the incident light to the LUMO level, which has the energy difference of 1.29 eV (Fig. 11). The second transfer path is from the Fermi level of Ag to the LUMO level of IC, which has the energy difference of 0.79 eV. The third transfer path is from the CB of ZnO to the LUMO of IC, which has an energy difference of 1.13 eV. ZnO is recognized as a typical n-type semiconductor, and in its nanostructure, there are plenty of surface oxygen vacancy defects that create surface-state energy levels. In ZnO, they are situated at 0.05 eV below the ZnO conduction band (CB) edge.^72^ Electrons in the defect levels of ZnO are excited by the incident light to surface-state energy levels with sub-bandgap energy and subsequently injected into the LUMO level of the IC molecules. The surface states of the intrinsic semiconductor are crucial to the charge transfer (CT) process from ZnO/Ag to the IC molecules, acting both as electron traps and intermediate states for electron transfer. A detailed investigation on the laser-wavelength-dependent SERS of the molecules adsorbed on the ZnO/Ag hybrid is considered for future studies.

The chemical enhancement mechanism effectively strengthens the Raman signal through multiple electron transfer processes.^73^ Additionally, the large surface area of ZnO NPls has the capacity to anchor more Ag NPs and adsorb more IC molecules on the SERS substrate. Consequently, ZnO/Ag possesses a large number of hotspots that serve as emitters of Raman signal. CM enhancement requires a direct bond between the molecules and the surface of the uneven ZnO/Ag protrusions. The large amount of adsorbed IC molecules benefits the resonant charge transfer between ZnO/Ag and the adsorbate.^74^ The CT process is primarily governed by the intrinsic properties of the adsorbed molecules and the surface characteristics of the semiconductor. The plentiful surface states of ZnO/Ag are favorable to the CT process and the enhancement of Raman signals. The interaction of IC molecules with the ZnO/Ag atoms *via* their nitrogen lone-pair electrons creates a strong bond between the adsorbed molecules and the SERS substrate surface. The Raman cross section is enormously enhanced, so a high sensitivity to probe surface and interface is obtained. Since the IC molecules are adsorbed strongly onto the ZnO/Ag nanostructure through the Lewis acid chemical bond, the chemical enhancement can be an important contributor. Decorating noble-metal nanoparticles on metal oxide semiconductor surfaces is an effective strategy for enhancing the Raman signal intensity.

## 4. Conclusions

In summary, we successfully fabricated a ZnO/Ag semiconductor/metal hybrid nanocomposite *via* hydrothermal and reduction routes for application in SERS. The ZnO/Ag nanomaterial exhibits enhanced Raman signals when indigo carmine dyes act as molecular probes. This work presents the observation of a SERS signal from IC molecules adsorbed on the ZnO NPl/Ag NP nanostructure for the first time. The limit of detection (LOD) was determined to be 5 × 10^−9^ M, and the enhancement factor (EF) was calculated to be 1.57 × 10^5^. The relationship between the logarithm of SERS intensity and the logarithm of IC concentration was linear in the range of 5 × 10^−9^ M to 10^−4^ M, and the uniformity was 3.6%. By comparing the SERS signals of pristine ZnO NPls, pure Ag NPs, glass substrate, and ZnO/Ag, an increase in Raman peak intensity was observed in the hybrid and could be ascribed to the combined effects of electromagnetic and chemical mechanisms, which are strongly influenced by the surface and interface properties. The EM mechanism is attributed to the contribution of Ag, while the CM or CT mechanism is ascribed to the contribution of ZnO. The synergistic effect of both mechanisms results in the SERS signal enhancement of IC adsorbed on the ZnO/Ag hybrid. The charge transfer (CT) mechanism from ZnO/Ag to IC molecules is influenced by the surface-state energy level of the ZnO NPls. Further, density functional theory (DFT) calculations revealed oscillations and electron transmission. This study holds significance in the realm of surface-enhanced Raman spectroscopy as it demonstrates the ability of semiconductor/metal nanostructures to support SERS signals. The efficacy of these nanostructures will facilitate the development of new high-performance SERS substrates. This work shows the potential application of metal oxide/noble metal nanostructures as SERS substrates beyond IC detection.

## Data availability

The datasets used and/or analyzed during the current study are available from the corresponding author upon reasonable request. In addition, all the data generated or analyzed during this study are included in this article.

## Author contributions

Ngo Thi Lan, Thi Thu Thuy Nguyen, Dong Thi Linh: investigation, DFT calculation, conceptualization, methodology; Nguyen Dac Dien, Xuan Hoa Vu: writing the original draft, visualization, and validation; Thi Thu Ha Pham, Truong Xuan Vuong, and Tran Thi Kim Chi: writing the original draft, supervision, reviewing, and editing; Pham Thi Nga, Tran Thi Huong Giang: data curation, resources; Tran Thu Trang, Nguyen Van Hao: data analysis, reviewing, and editing.

## Conflicts of interest

There are no conflicts to declare.
